# *Mycobacterium avium* Complex Pulmonary Disease as a Clinical Signal of Host Vulnerability in Advanced Myelofibrosis

**DOI:** 10.1002/ccr3.73538

**Published:** 2026-09-16

**Authors:** Mataichi Sekiya, Kalkidan Yekuno, Ishan Ghumare, Marina Margulis

**Affiliations:** ^1^ Department of Medicine NYC Health + Hospitals/South Brooklyn Health Brooklyn New York USA; ^2^ School of Medicine St. George's University St. George's Grenada

**Keywords:** *Mycobacterium avium*, myelofibrosis, opportunistic infection, ruxolitinib

## Abstract

An 82‐year‐old woman with JAK2‐mutated myelofibrosis who developed hemoptysis after initiation of ruxolitinib. She presented with anemia and ground‐glass opacities and was treated with antibiotics. She was readmitted with worsening respiratory symptoms. Sputum cultures yielded *Mycobacterium avium* . Her clinical course was complicated by leukocytosis with blasts prior to death.

## Key Clinical Message

The early onset of *Mycobacterium avium* Complex infection after ruxolitinib initiation may reflect underlying host immune vulnerability associated with advanced myelofibrosis, rather than being solely attributable to therapy‐induced immunosuppression.

## 1 Introduction

Ruxolitinib, a Janus kinase (JAK) 1/2 inhibitor, is widely used in the management of myelofibrosis and polycythemia vera and has been shown to improve symptoms and survival [1, 2]. However, JAK inhibition impairs cell‐mediated immunity and increases susceptibility to opportunistic infections [3].

Clinical studies have reported an increased risk of infections such as tuberculosis, cryptococcosis, and hepatitis B virus reactivation in patients receiving ruxolitinib [4]. In contrast, data on nontuberculous mycobacterial (NTM) infections remain limited.

In addition to treatment‐related immunosuppression, myelofibrosis itself is associated with immune dysregulation. Early disease stages are characterized by increased pro‐inflammatory cytokines, whereas advanced disease is associated with immunosuppressive cytokines, impaired T‐cell function, and reduced natural killer (NK) cell activity [5].

We report a case of rapidly progressive respiratory disease after ruxolitinib initiation, retrospectively considered consistent with *Mycobacterium avium* Complex (MAC) pulmonary disease in a patient with advanced myelofibrosis.

## 2 Case History/Examination

We report the case of an 82‐year‐old woman with a 19‐year history of JAK2‐mutated polycythemia vera/essential thrombocythemia. She had been treated with hydroxyurea and aspirin and had been followed regularly without major complications. She subsequently developed progressive constitutional and gastrointestinal symptoms, including poor appetite, diarrhea, abdominal discomfort, and weight loss, requiring hospitalization for suspected pancolitis. Approximately 1 month after hospitalization, she became transfusion‐dependent. Ruxolitinib was initiated 1 month later because of suspected progression to myelofibrosis.

## 3 Differential Diagnosis, Investigations, and Treatment

**FIGURE 1:**
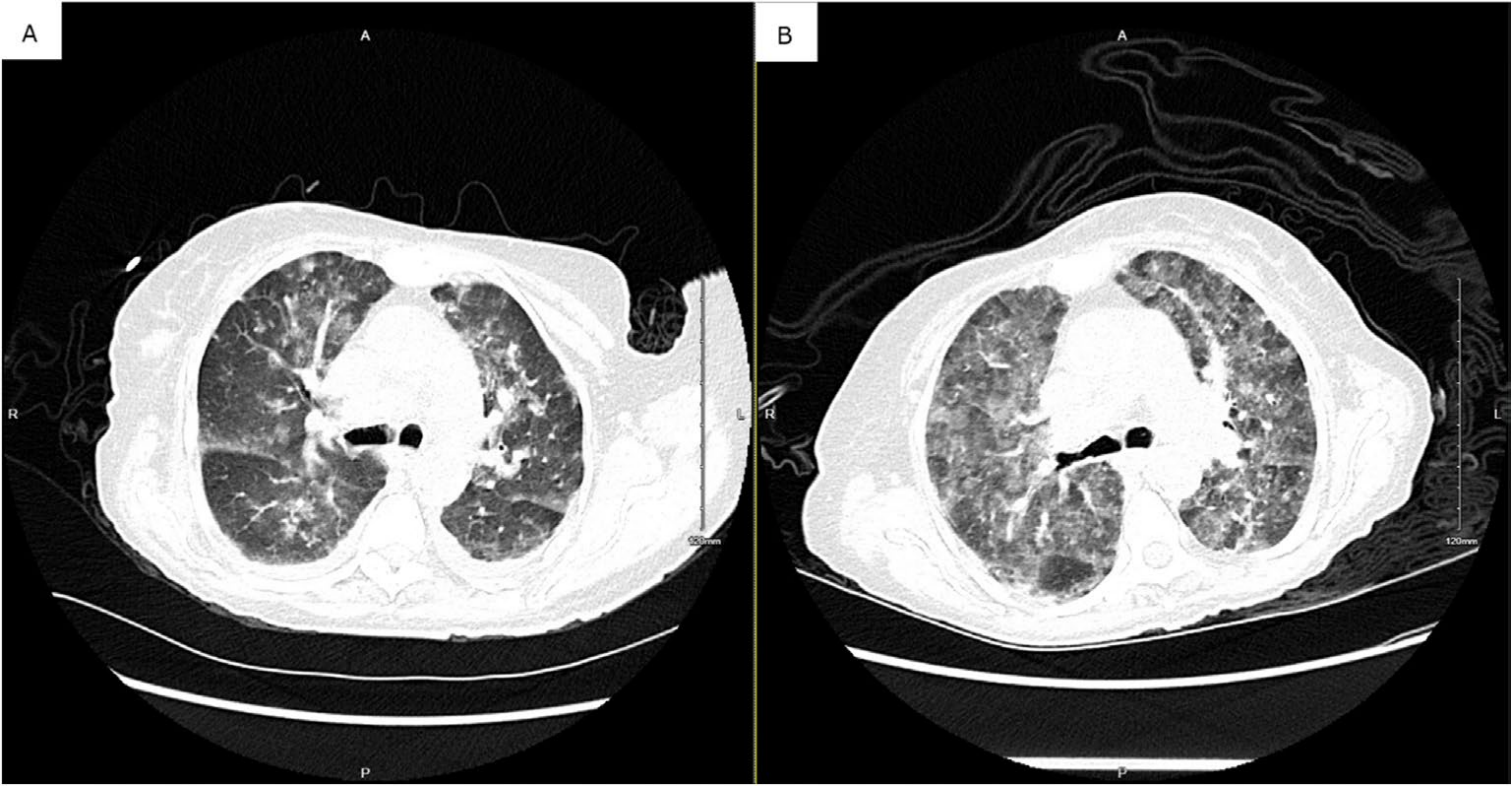
Chest computed tomography findings. (A) Chest computed tomography obtained during the initial hospitalization demonstrated patchy bilateral ground‐glass opacities. (B) Repeat chest computed tomography during the second hospitalization showed interval progression with more extensive bilateral ground‐glass opacities.

Approximately 3 months after the initiation of ruxolitinib, she was hospitalized for worsening hemoptysis and severe anemia (hemoglobin, 5.6 g/dL). Chest imaging showed patchy right lower lobe opacities with bilateral infiltrates, and computed tomography (CT) revealed bilateral ground‐glass opacities (Figure 1A). She was treated empirically with ceftriaxone and doxycycline for presumed community‐acquired pneumonia. An acid‐fast bacillus (AFB) culture of a sputum specimen obtained during this admission subsequently grew *M. avium* and *Mycobacterium chimaera* , which were identified by matrix‐assisted laser desorption/ionization time‐of‐flight (MALDI‐TOF) mass spectrometry. Antimicrobial susceptibility testing was not available. Given her clinical improvement, nonspecific radiographic findings, and isolation of multiple mycobacterial species from a single sputum specimen, these findings were interpreted as colonization.

Three weeks later, she was readmitted with recurrent hemoptysis and worsening dyspnea. CT demonstrated progression of the bilateral ground‐glass opacities (Figure 1B). Although she did not yet fully meet the diagnostic criteria for MAC pulmonary disease, the recurrence of respiratory symptoms and radiographic progression raised concern that the previously isolated *M. avium* and *M. chimaera* might be clinically significant. Azithromycin was therefore initiated empirically while further microbiologic evaluation was pending, and ruxolitinib was discontinued. Her respiratory symptoms initially improved, with resolution of fever, but she subsequently developed recurrent fever accompanied by clinical deterioration. Given concern for superimposed bacterial pneumonia, including possible anaerobic infection, antimicrobial therapy was broadened to piperacillin–tazobactam. Despite treatment, she developed progressive hypoxemia.

## 4 Conclusion and Outcome

On hospital Day 13, marked leukocytosis (26.3 × 10^3^/μL) with circulating blasts was noted, raising concern for leukemic transformation. Given her clinical deterioration, the patient was transitioned to comfort‐focused care and died on hospital Day 17. After her death, AFB cultures from two separate sputum specimens were confirmed to be positive for *M. avium* .

## 5 Discussion

Our case describes an 82‐year‐old woman with myelofibrosis who developed recurrent hemoptysis and progressive respiratory failure shortly after the initiation of ruxolitinib. During the initial hospitalization, a sputum culture grew *M. avium* and *M. chimaera* . However, the clinical significance of these isolates was initially uncertain because they were recovered from a single sputum specimen, the radiographic findings were nonspecific, and her symptoms improved with empirical antibacterial therapy. Three weeks later, recurrent respiratory symptoms and progressive radiographic abnormalities raised greater concern for clinically significant MAC infection. After her death, *M. avium* was confirmed in two separate sputum specimens, satisfying the microbiologic component of the diagnostic criteria for NTM pulmonary disease. In retrospect, the combination of progressive respiratory symptoms, radiographic deterioration, and repeated isolation of *M. avium* strongly suggested that she had developed MAC pulmonary disease during ruxolitinib therapy. Her clinical course was further complicated by progressive respiratory failure and suspected leukemic transformation, ultimately leading to death.

Antimicrobial management reflected the evolving clinical presentation and diagnostic uncertainty. During the initial hospitalization, ceftriaxone and doxycycline were administered empirically for presumed community‐acquired pneumonia because of the acute respiratory presentation and nonspecific radiographic findings. Given her clinical improvement and the initial isolation of two different NTM species from a single sputum specimen, guideline‐based multidrug therapy for MAC pulmonary disease was not initiated at that time. On readmission, recurrent hemoptysis, worsening dyspnea, and radiographic progression increased concern that the previously isolated *M. avium* and *M. chimaera* might be clinically significant, and azithromycin was initiated empirically while further microbiologic evaluation was pending. Ruxolitinib was also discontinued because of concern regarding treatment‐related immunosuppression. Although her respiratory symptoms initially improved with resolution of fever, she subsequently developed recurrent fever accompanied by clinical deterioration. Antibacterial therapy was therefore broadened to piperacillin–tazobactam for suspected superimposed bacterial pneumonia, including possible anaerobic infection. Confirmation of *M. avium* from two separate sputum specimens became available only after her death, precluding initiation of guideline‐based multidrug therapy for confirmed MAC pulmonary disease.

Ruxolitinib, a JAK1/2 inhibitor, is widely used in the treatment of myelofibrosis and polycythemia vera. While it provides significant clinical benefits, increasing concern has emerged regarding the risk of opportunistic infections due to Janus kinase/signal transducers and activators of transcription (JAK/STAT) pathway inhibition and subsequent impairment of cell‐mediated immunity [3]. In particular, there is accumulating evidence of tuberculosis in patients receiving ruxolitinib. However, reports of NTM infections, especially those caused by *M. avium* , remain limited. In the COMFORT‐II trial, only one case of tuberculosis was reported among 191 patients receiving ruxolitinib, and no cases of NTM infection were identified [6]. These findings may underestimate the true incidence of NTM infection, as enrollment from endemic regions was limited and systematic screening for NTM was not performed. In a systematic review comprising five Phase III randomized controlled trials, six Phase IV studies, and 28 case reports, a significant association was observed only between ruxolitinib and varicella–zoster virus reactivation [4]. Lescuyer et al. reported two cases of mycobacterial infection (one tuberculosis and one MAC infection) in a cohort of 65 patients with myelofibrosis or polycythemia vera treated with ruxolitinib. The MAC case involved disseminated infection after prolonged exposure (1410 days), with positive cultures from multiple sites and severe systemic manifestations [7]. Additional case reports have described MAC infection in the setting of pulmonary alveolar proteinosis associated with ruxolitinib [8, 9]. Notably, leukemic transformation was not observed in these prior reports.

In our case, several features suggest that factors beyond ruxolitinib‐induced immunosuppression may have contributed to susceptibility to and progression of MAC infection. The patient had constitutional symptoms, including appetite loss, diarrhea, and weight loss, before the initiation of ruxolitinib, which may have reflected underlying disease progression or, potentially, an unrecognized infection. Moreover, clinically apparent pulmonary disease occurred within approximately 3 months of starting ruxolitinib, substantially earlier than in previously reported cases. Compared with the cases reported by Lescuyer et al. [7] (1410 days), Sugiura et al. [8] (approximately 2 years), and El Kik et al. [9] (more than 1 year), the rapid clinical progression in our patient suggests that preexisting host vulnerability may have played an important role.

Multiple mechanisms of immunodysregulation in myelofibrosis may contribute to susceptibility to MAC infection. Host defense against MAC relies primarily on the interleukin‐12 (IL‐12)/interferon‐γ (IFN‐γ) axis. Macrophages infected with MAC produce IL‐12, which stimulates IFN‐γ production from T‐cells and natural killer (NK) cells. IFN‐γ, in turn, activates macrophages to eliminate intracellular pathogens through enhanced phagolysosomal activity and granuloma formation [10, 11]. In patients with myelofibrosis, several abnormalities may impair this pathway. Reduced Th1 cell function leads to decreased IFN‐γ production, while increased expression of immune checkpoint molecules such as programmed cell death‐1 (PD‐1) and cytotoxic T lymphocyte antigen‐4 (CTLA‐4) further suppresses T‐cell activity [12, 13]. Additionally, NK cell dysfunction, including impaired maturation and increased inhibitory receptor expression, has been reported [14]. These alterations may significantly compromise host defense against MAC infection.

In addition, her persistent anemia despite transfusion, constitutional symptoms before treatment, and rapid progression to suspected leukemic transformation suggest that her myelofibrosis may already have been at an advanced stage at the time of ruxolitinib initiation. Therefore, disease‐related immune dysregulation may have played a major role in her susceptibility to and progression of MAC infection, in addition to the immunosuppressive effects of ruxolitinib.

In conclusion, this case suggests that clinically significant MAC infection occurring shortly after the initiation of ruxolitinib may reflect underlying immune vulnerability associated with advanced myelofibrosis, in addition to treatment‐related immunosuppression. Although MAC pulmonary disease was not definitively established during the patient's lifetime, the subsequent repeated isolation of *M. avium* , together with progressive respiratory symptoms and radiographic abnormalities, strongly supported this diagnosis retrospectively. Clinicians should consider both disease‐related immunodeficiency and treatment‐related immunosuppression when evaluating opportunistic infections in patients with myelofibrosis receiving ruxolitinib.

## Author Contributions

**Mataichi Sekiya:** writing – original draft. **Kalkidan Yekuno:** writing – original draft. **Ishan Ghumare:** writing – original draft. **Marina Margulis:** supervision, writing – review and editing.

## Funding

The authors have nothing to report.

## Ethics Statement

According to our institutional policy, ethics approval was not required for this case report, and the institutional ethics committee approved its publication.

## Consent

The patient died before this case report was prepared. All identifiable information was removed to ensure patient anonymity and written informed consent was obtained from the patient's daughter.

## Conflicts of Interest

The authors declare no conflicts of interest.

## Data Availability

The data that support the findings of this study are available on request from the corresponding author. The data are not publicly available due to privacy or ethics restrictions.
